# Longitudinal monitoring of circulating tumor cell dynamics for potential prediction of early recurrence and clinical outcomes after curative resection of hepatocellular carcinoma: a pilot study

**DOI:** 10.1186/s12885-026-15638-7

**Published:** 2026-01-29

**Authors:** Dhruvajyoti Roy, Olaf Guckelberger, Elsie Oppermann, Ibrahim Büdeyri, Roxana Chaikhoun, Natascha Kohl, Michel Kostantin, Darius Zokai, Matthias Knaak, Shadi Katou, Felix Becker, Andreas Andreou, Haluk Morgul, Benjamin Struecker, Vladimir P. Zharov, Andreas Schnitzbauer, Thomas J. Vogl, Wolf O. Bechstein, Andreas Pascher, Markus S. Zimmermann, Mazen A. Juratli

**Affiliations:** 1 Department of Breast Surgical Oncology, The University of Texas M. D. Anderson Cancer Center, Houston, TX United States of America; 2 Department of General, Visceral and Transplant Surgery, Muenster University Hospital, Muenster, Germany; 3 Department of General, Visceral and Transplant Surgery, Frankfurt University Hospital, Goethe University, Frankfurt am Main, Germany; 4 Department of Psychiatry and Psychotherapy, University Medical Centre Hamburg- Eppendorf, Hamburg, Germany; 5 Arkansas Nanomedicine Center, University of Arkansas for Medical Sciences, Little Rock, Arkansas, United States of America; 6 Department of Visceral, Oncological, and Transplant Surgery, University Hospital Knappschaftskrankenhaus, Bochum, Germany; 7 Clinic for Radiology and Nuclear Medicine, Frankfurt University Hospital, Goethe University, Frankfurt am Main, Germany; 8 Clinic for General and Visceral Surgery, Schön Clinic Neustadt, Neustadt in Holstein, Germany

**Keywords:** Early detection of cancer, Hepatocellular carcinoma (HCC), Cancer biomarkers, CTCs, Liquid biopsy, Recurrence

## Abstract

**Background:**

Hepatocellular carcinoma (HCC) has a high recurrence rate even after curative hepatectomy. Circulating tumor cells (CTCs) have emerged as promising liquid biopsy biomarkers for minimal residual disease and early recurrence. This study evaluated longitudinal CTC dynamics and their prognostic significance in HCC patients undergoing curative resection.

**Methods:**

We prospectively analyzed CTCs in 27 HCC patients at four time points: preoperatively, immediately postoperatively, and at 6 and 12 months after surgery. CTCs were defined as CD45⁻/CD146⁺/ASGPR⁺ using multicolor flow cytometry and immunofluorescence. Recurrence-free survival (RFS) and overall survival (OS) were assessed during a median 3.7-year follow-up. Control groups included 29 patients with non-malignant or non-HCC liver tumors and 8 healthy donors.

**Results:**

Preoperative CTCs were detected in 48.2% of HCC patients (mean 0.46 cells/mL). Detection rates rose to 95% at 6 months and 100% at 12 months post-surgery. Persistent or rising postoperative CTCs were strongly associated with early recurrence (51.8% overall) and reduced RFS (*p* = 0.02) and OS (*p* = 0.05). No significant correlation was observed between CTC levels and tumor size or volume, AFP, or IL-6 levels.

**Conclusions:**

Longitudinal CTC monitoring provides an early and non-invasive indicator of recurrence risk and survival after curative HCC resection. Persistent CTCs may represent minimal residual disease and a potential therapeutic target for improving long-term outcomes.

**Supplementary Information:**

The online version contains supplementary material available at 10.1186/s12885-026-15638-7.

## Introduction

Hepatocellular carcinoma (HCC) is one of the most prevalent cancers globally and ranks as the third leading cause of cancer-related deaths [1]. Although surgical resection is the standard curative treatment for HCC, the overall prognosis remains poor, with a 5-year survival rate as low as 12.7% [2]. This unfavorable prognosis is largely due to the high rates of recurrence and metastasis following resection. Most patients experience relapse within the first few years after surgery, often due to undetected residual tumor cells. The lack of sensitive and precise biomarkers for detecting early metastasis and recurrence often results in delayed interventions, leaving patients with missed opportunities for curative treatment. Consequently, the long-term survival rates for HCC patients remain dismal, underscoring the need for novel biomarkers to improve prognostication and guide early intervention [3].

Emerging evidence suggests that even in cases where HCC is treated early, residual cancer cells can persist in the bloodstream, leading to tumor recurrence and, in some cases, more aggressive disease progression. Circulating tumor cells (CTCs), which are shed from the primary tumor into the bloodstream, have shown considerable potential as biomarkers for the early detection of cancer recurrence and metastasis. Measuring and molecularly subtyping CTCs can provide critical insights into tumor biology, offering a non-invasive method to monitor disease progression and predict recurrence-free survival (RFS) and overall survival (OS) in HCC patients [4–7]. The detection of CTCs, particularly in the post-operative period, could help identify patients at higher risk of recurrence, enabling timely therapeutic interventions.

In the current study, we aimed to evaluate the clinical significance of CTCs in HCC patients who underwent curative liver resection. We hypothesized that the presence of CTCs, particularly in the post-operative period, could serve as a critical predictor of recurrence and metastasis. The detection of CTCs would likely correlate with adverse clinical outcomes, including reduced RFS and OS. To test this hypothesis, we used standardized fluorescence-activated cell sorting (FACS) and immunofluorescence (IF) microscopy to explore the prevalence, dynamic changes, and prognostic significance of these cells in HCC patients undergoing curative resection in a prospective manner. By examining the presence of CTCs before and after surgery, we sought to determine their potential role in predicting long-term outcomes and guiding post-operative management strategies in HCC patients.

## Materials and methods

### Study population

Our prospective single-center study, conducted from August 2016 to July 2018 at Frankfurt University Hospital, enrolled 56 patients and 8 heathy volunteers. This patient cohort included 27 HCC patients scheduled for liver resection, 13 patients with non-malignant liver disease (NMLD), and 16 patients with tumors other than HCC. For the HCC group, patients were excluded if they had liver metastases of extrahepatic origin, unresectable intrahepatic disease with major vessel involvement, concurrent HIV infection, or severe comorbidities. We employed the Barcelona Clinic Liver Cancer (BCLC) criteria for tumor staging of the HCC patients, while tumor grading was assessed using the Edmondson grading system describing histological differentiation. While the BCLC guidelines typically recommend that patients with BCLC B/C stage are not suitable for surgical resection, our inclusion criteria allowed surgical intervention after multidisciplinary-team assessment, if liver function was preserved (Child–Pugh A), future liver remnant volume was adequate, and no extrahepatic disease was present. For patients with multiple lesions (BCLC B stage), “tumor size” refers to the diameter of the largest lesion, consistent with BCLC criteria.

### Study design and ethical considerations

**Fig. 1:**
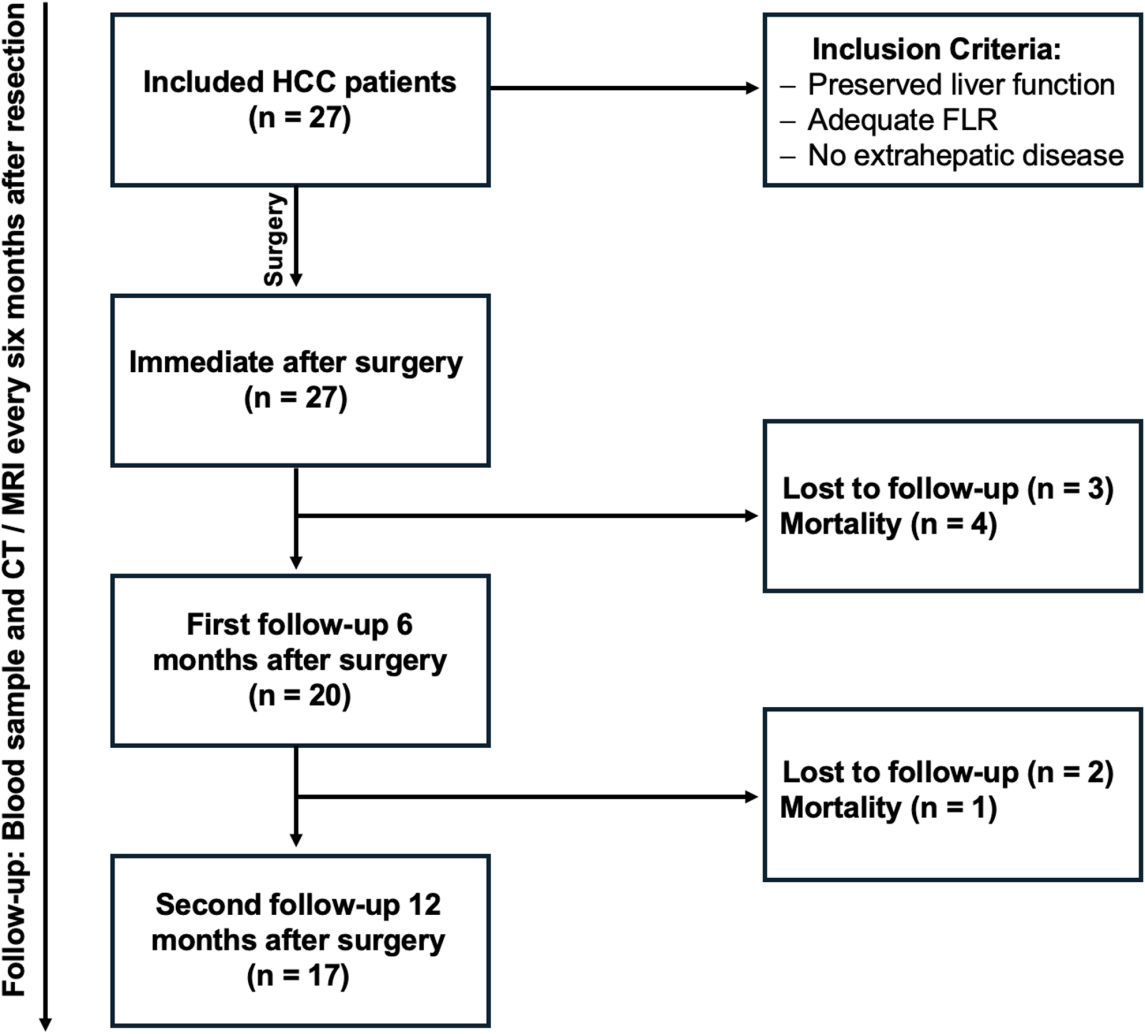
Flowchart outlining the study design, patient enrollment and follow-up process. *HCC* Hepatocellular carcinoma, *FLR* Future liver remnant

Blood samples were collected from 27 HCC patients at four distinct time points: immediately prior to tumor resection (baseline), shortly after the procedure, and during follow-up appointments at 6 and 12 months after surgery. The patients’ clinical progress was monitored for recurrence every six months after resection via CT or MRI imaging for a median follow-up of 3.7 years (2.5–4.3 years). Furthermore, no patient received adjuvant therapy following tumor resection. To improve the clarity of our methodology and patient progression throughout the study, we have included a flowchart (Fig. 1) that illustrates the patient enrollment process, the timeline of blood sample collection, and the follow-up schedule for monitoring recurrence.

Due to various factors, including tumor-related mortality and study discontinuation, the number of participants decreased to 20 by the time of the first follow-up and 17 by the time of the second follow-up. For HCC patients, baseline blood samples were obtained intraoperatively from the central venous catheter just before and on the same day following tumor removal. Subsequent samples were collected as part of routine post-operative care and tumor surveillance. Blood samples from patients with NMLD and tumors other than HCC were also collected before and after their respective surgeries, though no follow-up or extended observation period was implemented for these groups. Healthy donors provided a single blood sample. All blood specimens were collected in EDTA-tubes (Sarste, Nümbrecht, Germany).

The study was conducted in accordance with the Declaration of Helsinki [8] and approved by the Ethics Committee of the University of Frankfurt’s ethics committee (approval number: 312/16). Written informed consent was obtained from all participants prior to data collection and analysis. The study was registered in the German Clinical Trials Register (DRKS) under the clinical trial number: DRKS00034959, registration date: August 26, 2024. This study was reported in accordance with the Strengthening the Reporting of Observational Studies in Epidemiology (STROBE) guidelines.

### Enrichment and detection of CTCs

For each participant, a 7.5 mL blood sample was obtained. Peripheral blood mononuclear cells (PBMCs) were isolated using density gradient centrifugation with OncoQuick^®^ tubes (Greiner Bio-One, Kremsmünster, Austria). The process involved carefully layering the blood sample over a pre-cooled filter in the OncoQuick^®^ tube, followed by centrifugation at 1600 x g and 4 °C for 20 min. The isolated cells underwent two washing steps using phosphate-buffered saline (PBS; Gibco/Invitrogen, Karlsruhe, Germany) without calcium and magnesium ions, supplemented with 0.5% bovine serum albumin (BSA; Sigma‒Aldrich Chemie GmbH, Munich, Germany). The washed cells were then transferred to flow cytometry tubes. A cocktail of fluorescently-labeled antibodies, including anti-human Asialoglycoprotein Receptor 1 antibody (ASGPR-1)-PE (BD Pharmingen, Heidelberg, Germany), anti-human CD146 antibody (also called Melanoma Cell Adhesion Molecule, MCAM-APC; clone: SHM-57 (BioLegend, San Diego, CA, USA), and anti-CD45-FITC; clone: HI30 (BD Pharmingen), was applied to the cells. The antibody incubation was carried out at 4 °C for one hour. Following incubation, excess antibodies were removed by two additional washing steps with PBS containing 0.5% BSA. The prepared samples were analyzed using a four-laser FACSAria Fusion flow cytometer (BD Biosciences, CA, USA) in conjunction with FACSDiva Software (version 8.0.1). CTCs were identified based on their characteristic immunophenotype: CD45-negative, CD146-positive, and ASGPR-positive (Supplementary Fig. 1). This protocol was developed through previous research conducted by our group, as reported by Vogl et al. [9]. To assess the sensitivity and specificity of the selected antibodies, preliminary trials were performed using HepG2 cell lines (obtained from Cell Lines Service GmbH, Eppelheim, Germany) and blood samples from healthy donors.

### End points

The primary end point was OS which defined as the time between the date of surgery and death from any cause or last follow-up. Secondary end points included RFS, CTC counts, tumor volume and size, AFP levels, IL-6 levels, microvascular invasion (MVI), tumor-free resection margin (R0). RFS was defined as the interval between surgery and recurrence detection via imaging (CT or MRI). For patients without recurrence or death by the study’s end, RFS and OS were censored at the last follow-up date. We defined early recurrence as tumor recurrence within 12 months following surgical resection keeping in line with the literature [10–13]. A threshold of ≥ 1 CTC/7.5 mL, which has been shown to be prognostic in a number of studies, such as in primary breast cancer, including locally advanced and inflammatory breast cancer [14, 15], was used for our analysis at each of the blood draw time points.

### Statistical analysis

Patient follow-up continued through September 2019, with medical data obtained from electronic health records. Statistical analyses were conducted using R version 4.0.3 (R Foundation for Statistical Computing, Vienna, Austria) and GraphPad Prism version 8.0 (La Jolla, CA, USA). Survival curves were generated using the Kaplan-Meier method and compared with the univariate log-rank test. An unpaired t-test with Welch’s correction assessed differences in data distributions. Descriptive statistics summarized categorical variables as counts and percentages, while continuous variables were reported as means and standard deviations if normally distributed, or medians and interquartile ranges otherwise. Statistical significance was evaluated using various methods: Fisher’s exact test for categorical variable independence, Mann-Whitney U tests for non-normally distributed continuous variables, two-sample Z tests for proportion comparisons, and log-rank tests for survival analyses. All p-values were two-sided, with significance set at *p* < 0.05. To account for multiple comparisons across the three groups, p-values were adjusted using the Holm method. This comprehensive statistical approach allowed for robust analysis of the diverse data collected in this study.

## Results

### Patient characteristics

The study included a total of 56 patients across three groups. Table 1 provides a comprehensive overview of the demographic, clinical, and pathological characteristics of the HCC patient group. Table 2 delineates a comparative distribution of CTCs among 56 patients. The HCC group comprised the largest portion with 27 patients (42.2%), followed by 16 patients (25%) with tumors other than HCC, and 13 patients (20.3%) with NMLD. Gender distribution varied among the groups, with males predominating in the HCC group (74.1%) and the other tumor group (62.5%), while females were more prevalent in the NMLD group (69.2%). The mean age was similar across all groups, with HCC and NMLD patients averaging 62 years and patients with other tumors averaging 61 years. The age range was widest for the HCC group (28–83 years), followed by the NMLD group (23–75 years), and the other tumor group (47–85 years). These demographic characteristics provide a baseline understanding of the patient population involved in the study, highlighting the gender disparities and age distributions across the different liver-related conditions. Table 1 summarizes the clinical demographics and tumor characteristics of the 27 patients with HCC enrolled in our study. The median age of patients was 65 ± 14.8 years. Most patients were male (74.1%, *n* = 20). Regarding liver parenchymal texture, 44.5% (*n* = 12) had liver cirrhosis, while 22.3% (*n* = 6) presented with liver fibrosis. Metabolic-associated steatohepatitis (MASH) was observed in 14.9% (*n* = 4) of patients. Comorbidities included diabetes mellitus in 40.7% (*n* = 11) of patients. Etiological factors such as alcohol abuse and hepatitis virus infection were present in 25.9% (*n* = 7) and 37% (*n* = 10) of patients, respectively. According to the BCLC staging, 51.8% (*n* = 14) were classified as stage 0 or A, while 48.2% (*n* = 13) were stage B or C. Five BCLC B/C patients received preoperative down-staging therapy to enable resection. The mean tumor volume was 171.00 ± 825.30 cm³, with 59.3% (*n* = 16) of tumors larger than 5 cm. Most surgeries (85.1%, *n* = 23) were performed using an open approach. Preoperative alpha-fetoprotein (AFP) levels were elevated (> 7.0 ng/mL) in 59.3% (*n* = 16) of patients. Histopathologically, 85.1% (*n* = 23) of tumors were grade I or II, and microvascular invasion was observed in 29.7% (*n* = 8) of cases. Tumor-free resection margins were achieved in 85.1% (*n* = 23) of surgeries. Post-operatively, recurrence and mortality rates were both 51.8% (*n* = 14).

**Table 1:** Clinical characteristics and pathological data of the HCC patients. *IQR* Interquartile range

Clinical characteristics	No. of patients (%)
Age, years	Mean: 62 ± 14.8;
	Median (IQR): 65.50 (56.50, 72.25)
Sex	
Male	20 (74.1)
Female	7 (25.9)
Liver cirrhosis	
No	15 (55.5%)
Yes	12 (44.5%)
Liver fibrosis	
No	21 (77.7%)
Yes	6 (22.3%)
MASH	
No	23 (85.1%)
Yes	4 (14.9%)
Diabetes mellitus	
No	16 (59.3%)
Yes	11 (40.7%)
Alcohol abuse	
No	20 (74.1)
Yes	7 (25.9)
Hepatitis virus infection	
No	17 (63.0%)
Yes	10 (37.0%)
BCLC stage	
0 + A	14 (51.8%)
B + C	13 (48.2%)
Tumor volume in cm³	
Mean (± std)	460.77 (± 825.30)
Median (IQR)	171.00 (18.91, 353.25)
Tumor size, cm	
≤ 5	11 (40.7%)
> 5	16 (59.3%)
Preoperative therapy	
No	22 (81.0%)
Yes	5 (19.0%)
Type of preoperative therapy	
Transarterial chemoembolization	4 (80.0%)
Microwave ablation	1 (20.0%)
Operation method	
Open	23 (85.1%)
Laparoscopic	4 (14.9%)
Resection type	
Segmental liver resection	17 (63%)
Hemihepatectomy	10 (37%)
IL-6, pg/mL	
Negative (≤ 7.0)	17 (63%)
Positive (> 7.0)	10 (37%)
AFP, ng/mL	
Negative (≤ 7.0)	11 (40.7%)
Positive (> 7.0)	16 (59.3%)
Histological Grading	
I, II	23 (85.1%)
III	4 (14.9%)
Microvascular invasion	
No	19 (70.3%)
Yes	8 (29.7%)
Tumor-free resection margin	
No	4 (14.9%)
Yes	23 (85.1%)
Recurrence	
No	13 (48.2%)
Yes	14 (51.8%)
Site of recurrence	
Intrahepatic	11 (80.0%)
Extrahepatic	3 (20.0%)
Mortality	
No	13 (48.2%)
Yes	14 (51.8%)
Cause of death	
HCC-related	14 (100.0%)
Non-HCC-related	0 (0.0%)
CTC/ml before resection	
Negative	14 (51.8%)
Positive	13 (48.2%)
CTC/ml 1 day after surgery	
Negative	14 (51.8%)
Positive	13 (48.2%)
CTC/ml 6 months after surgery	
Negative	1 (5%)
Positive	19 (95%)
CTC/ml 12 months after surgery	
Negative	0 (0%)
Positive	17 (100%)

**Table 2:** Perioperative distribution of CTCs among study patients. HCC: hepatocellular carcinoma, NMLD: nonmalignant liver disease

	HCC *n* (%)	NMLD *n* (%)	Tumor other than HCC *n* (%)
Total (*n* = 56)	27 (42.2)	13 (20.3)	16 (25.0)
Gender			
Female (%)	7 (25.9)	9 (69.2)	6 (37.5)
Male (%)	20 (74.1)	4 (30.8)	10 (62.5)
Age (years)			
Mean	62	62	61
Range	28–83	23–75	47–85
CTC/ml before surgery			
Mean	0.46	0.02	0.025
Range	0–2.90	0–0.27	0–0.27
CTC/ml immediately after surgery			
Mean	0.51	0.03	0.019
Range	0–4.00	0–0.40	0–0.13

### CTC enumeration and distribution according to clinical-pathological characteristics

**Fig. 2:**
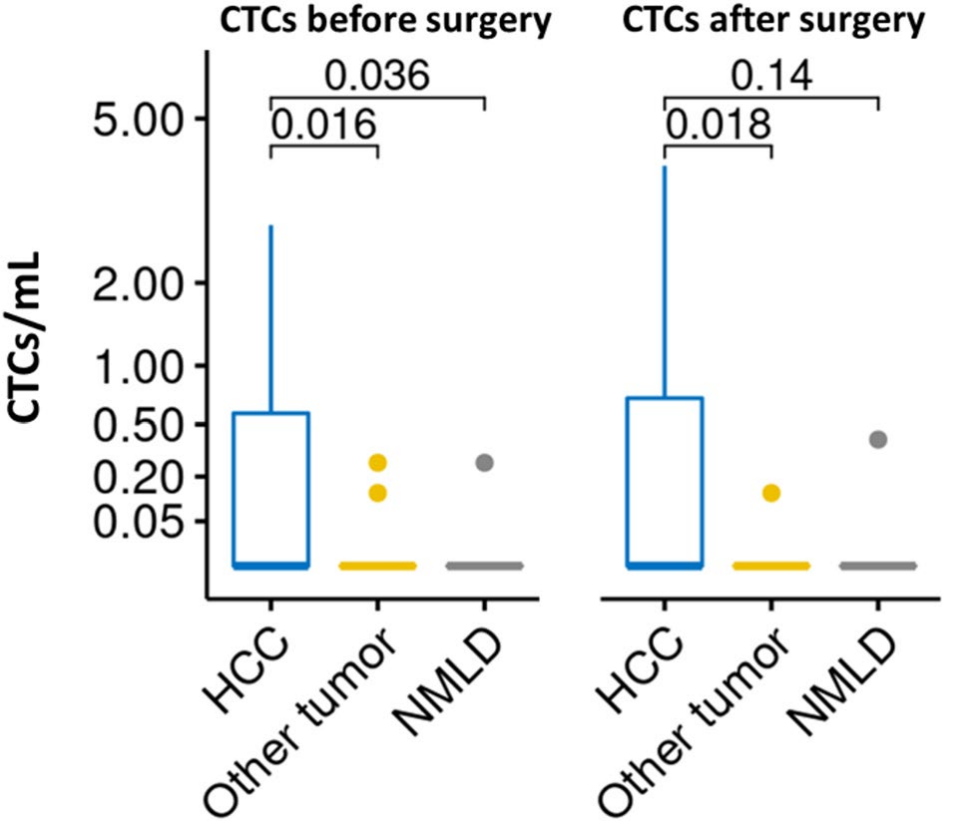
Comparison of CTC counts [before surgery (left) and after surgery (right)] in patients with HCC, NMLD, and other tumor types

CTCs were detected in 48.2% (*n* = 13) of patients before surgery, with detection rates of 48.2% (*n* = 13), 95% (*n* = 19), and 100% (*n* = 17) at immediately after surgery, 6 months, and 12 months after surgery, respectively. In contrast, only 3 individuals from the control group (*n* = 36) exhibited CTC positivity, comprising one patient with NMLD and two with non-HCC cancers. The mean CTC count per milliliter of blood for each group before and immediately after surgery is presented in Table 2. In addition, Fig. 2 provides a visual representation of CTC distribution across patient groups (HCC, NMLD, and other cancer types) before and immediately after surgical intervention. Statistical analysis revealed that HCC patients had significantly higher CTC counts compared to both NMLD (*p* = 0.036) and other tumor types (*p* = 0.016) before surgery. After surgery, the difference remained significant for other cancer types (*p* = 0.018) but not for NMLD (*p* = 0.14). These findings highlight distinct CTC patterns among different patient cohorts and at various timepoints.

### Longitudinal evaluation of CTCs detected in HCC patient samples

**Fig. 3:**
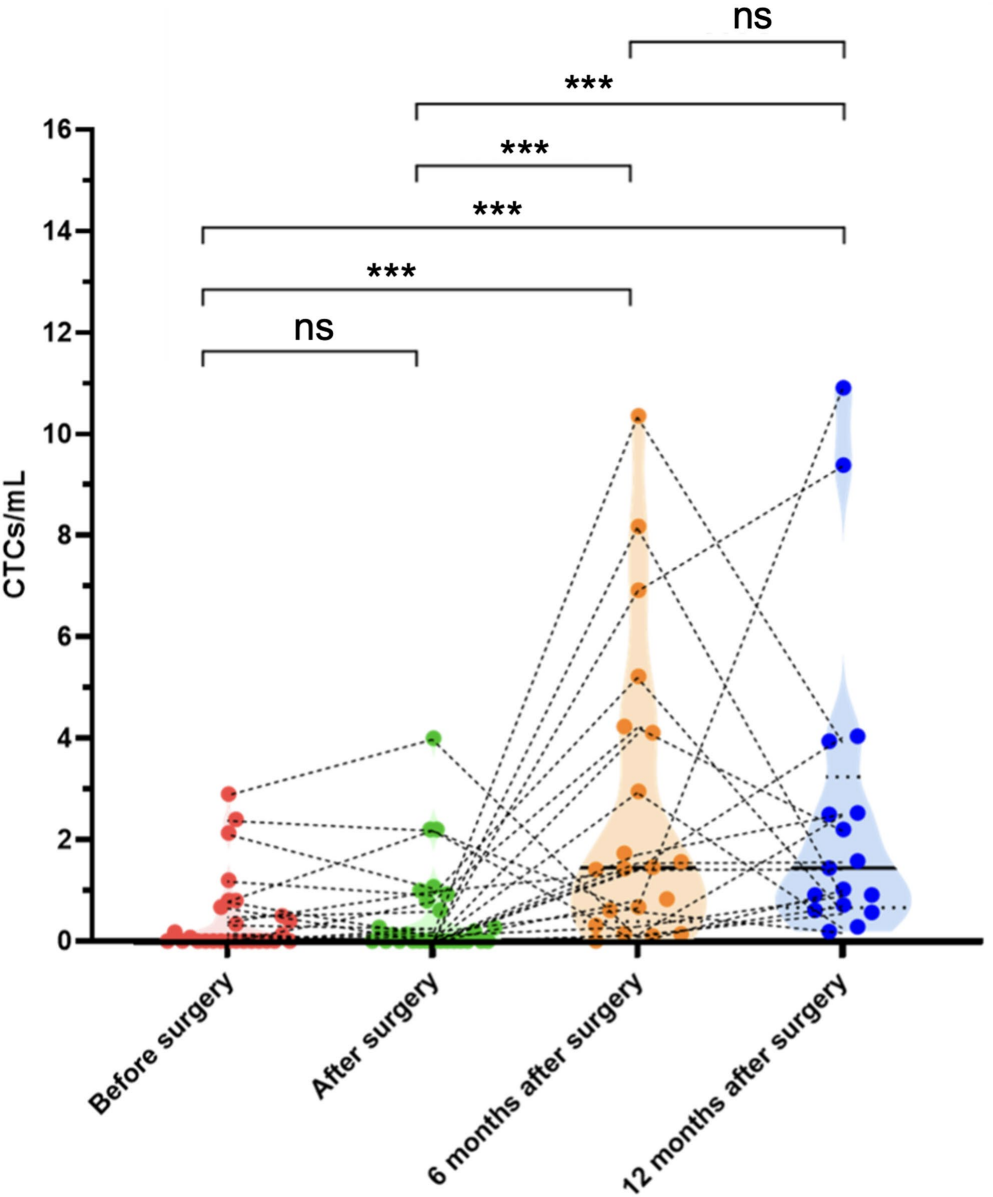
Dynamics of CTCs before and after surgery. Violin plots depict the distribution of CTC counts at four time points: before surgery (red), immediately after surgery (green), 6 months after surgery (orange), and 12 months after surgery (blue). Each dot represents an individual patient, and black dotted lines connect serial measurements within the same patient to illustrate longitudinal changes. The width of each violin reflects the kernel density estimation of CTC values. Notable increases in CTCs were observed at 6 months after surgery, with statistically significant differences compared with before surgery (p = 0.0031), immediately after surgery (p = 0.0040), and 12 months (p = 0.83). CTC counts at 12 months remained elevated compared to before surgery and immediately after surgery (p = 0.0066 and p = 0.0084 respectively) but were lower than at 6 months. P-values from pairwise comparisons (Wilcoxon signed-rank test) are shown above the plot. *ns* not significant, *** p< 0.001

In total, 27 HCC patients were enrolled in this study and the sequential analysis of the CTCs from the patients at the baseline and immediately after surgery, and at 6 and 12 months after surgery are shown in Fig. 3. The volcano plots depict the distribution of total CTCs/mL before surgery, immediately after surgery, and at 6 and 12 months after surgery. This visualization allows for the tracking of CTC dynamics over time, potentially revealing patterns in CTC levels that could be indicative of disease progression or treatment response. Of the 13 patients who were preoperatively CTC-positive, 10 (76.9%) remained CTC-positive immediately after surgery. This trend continued, with 9 out of these 10 patients (90%) remaining CTC-positive at 6 months and 8 out of 9 (88.9%) at 12 months after surgery.

### Short-term dynamic of CTCs

Short-term CTC dynamics in HCC patients showed minimal changes immediately following surgery. The mean CTCs/mL before surgery was 0.46 ± 0.80, with a median of 0.00 (IQR: 0.00, 0.58). Immediately after surgery, there was a slight increase in CTCs/mL, with a mean of 0.51 ± 0.94 and a median of 0.00 (IQR: 0.00, 0.70). There is no significant difference between the CTC-counts pre- vs. immediately after the surgery (*p* = 0.96).

### Long-term dynamic of CTCs

Long-term CTC dynamics revealed a substantial increase in CTCs at both 6 and 12 months after surgery. At 6 months, the mean CTCs/mL rose significantly to 2.31 ± 2.60, with a median of 1.45 (IQR: 0.75, 2.34). This trend continued at 12 months after surgery, with a mean of 2.15 ± 2.49 CTCs/mL and a median of 1.44 (IQR: 0.97, 1.89). Longitudinal evaluation of CTCs detected in HCC patient samples demonstrated a clear pattern of increase over the 12-month follow-up period. As illustrated in Fig. 3, the violin plots show the distribution of total CTCs/mL at each time point, with a notable upward trend from the baseline before surgery to the 6- and 12-month follow-ups (*p* = 0.0031 and 0.0066 respectively). Significant differences were observed from immediately after surgery to the 6- and 12-month follow-ups as well (*p* = 0.0040 and 0.0084 respectively). However, no significant differences were observed from the 6-month to the 12-month follow up (*p* = 0.83).

### Prognostic value of CTCs and tumor size on survival outcomes

**Fig. 4:**
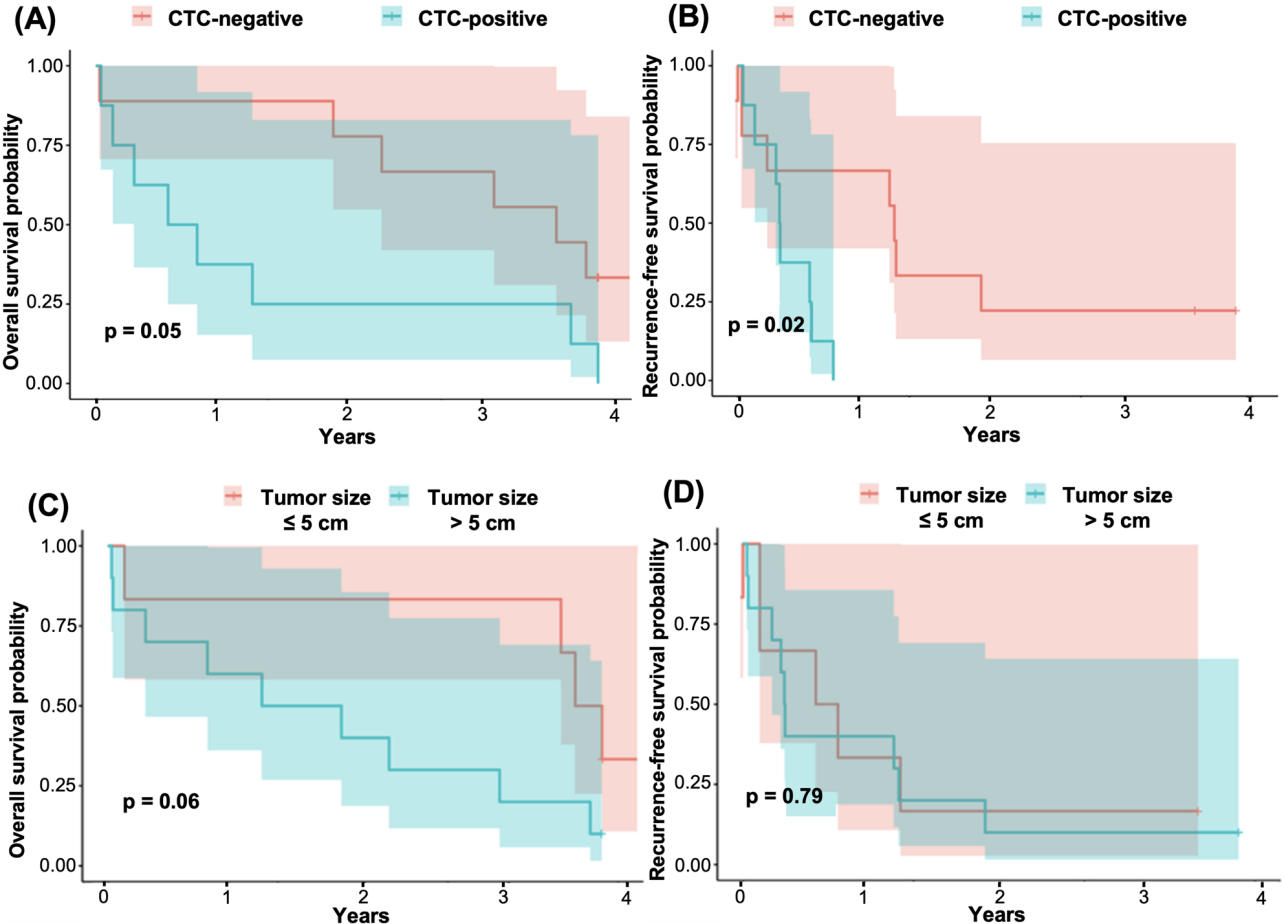
Prognostic value of immediate postoperative CTC positivity and tumor size on survival outcomes. Kaplan–Meier survival curves depict OS and RFS stratified by immediate postoperative CTC status (Panels A–B) and tumor size (Panels C–D). **A** Patients with CTC-positive status (cyan) exhibit lower OS compared to CTC-negative individuals (pink), with a marginally significant difference (p= 0.05). **B** CTC-positive patients also have significantly worse RFS than CTC-negative patients (p = 0.02), especially within the first-year follow-up. **C** Patients with tumors ≤ 5 cm (pink) show a non-significant trend toward better OS compared to those with tumors > 5 cm (cyan) (p = 0.06). **D** No significant difference is observed in RFS between patients with tumor sizes≤ 5 cm and > 5 cm (p = 0.79)

Prognostic significance of immediate postoperative CTC status and tumor size in HCC patients are analyzed with log rank test and depicted through Kaplan-Meier curves (Fig. 4). The curves compare OS and RFS between CTC-positive and CTC-negative groups, as well as based on tumor size (> or ≤ than 5 cm). The univariate analysis reveals that CTC positivity is a statistically significant independent predictor of adverse outcomes in hepatocellular carcinoma patients, with CTC-positive status associated with significantly reduced OS (*p* = 0.05) and RFS (*p* = 0.02), whereas tumor size alone fails to demonstrate significant prognostic value for either OS (*p* = 0.06) or RFS (*p* = 0.79) in this surgical cohort. We found that HCC patients with higher CTC levels immediately after the surgery (cut-off ≥ 1 CTC/7.5 mL) had a significantly lower OS probability (Fig. 4 A) and a significantly lower RFS probability (Fig. 4B). HCC patients with larger tumors demonstrated a divergent trajectory toward poorer OS and RFS, although statistical significance was not achieved (Fig. 4 C and D, respectively).

### Correlation between CTCs and key tumor parameters

We also investigated potential relationships between the CTC level before surgery and two key tumor parameters: tumor size and resected tumor volume (Supplementary Fig. 2 A and 2B). This correlation plots aimed to determine whether CTC counts before surgery could serve as indicators of tumor burden or extent of disease in HCC patients. However, upon examination of these plots, no clear correlations were observed between CTC levels before surgery and either tumor size or resected tumor volume.

### Correlation between CTCs and typical HCC biomarkers

We also explored the potential associations between CTC levels and established HCC biomarkers, namely AFP (alpha-fetoprotein) and IL-6 (interleukin-6). The analysis reveals no significant correlation between CTCs/mL and either AFP or IL-6 levels. The scatter plot analyses in Supplementary Fig. 3 revealed minimal to no consistent correlation between AFP levels and CTC counts at all observed time points (immediately, 6 months, and 12 months after surgery). Although several patients demonstrated extremely high AFP levels (e.g., > 15,000 and > 60,000 ng/mL), these did not correspond with elevated CTC counts, indicating a lack of direct relationship. In contrast, IL-6 showed a more comparable pattern in relationship to CTC levels. At 6 months, a cluster of patients with IL-6 levels between 5 and 10 pg/mL exhibited moderately elevated CTCs. Similarly, at 12 months, a spike in CTC count was observed in patients with IL-6 levels in the 1.3–2.3 pg/mL range. Further, AFP and IL-6 levels in relation to CTC counts at three time points after surgery revealed distinct temporal patterns (Supplementary Fig. 4). Immediately after surgery, no clear relationship was observed, with most patients exhibiting low CTC counts regardless of AFP or IL-6 levels. At 6 months, a trend emerged where elevated IL-6 levels (≥ 5 pg/mL) in conjunction with rising AFP were associated with markedly increased CTC counts (up to 10 CTCs/mL). By 12 months, although AFP and IL-6 values were generally lower, isolated cases still showed modest CTC elevations, particularly in those with moderate IL-6 and AFP levels.

### Impact of surgical approaches on CTC dynamics

We also investigated the impact of surgical approach on CTC levels comparing laparoscopic and open approach revealing distinct temporal CTC temporal dynamics (Supplementary Fig. 5). Specifically, patients who underwent laparoscopic surgery exhibited a notable increase in CTC levels at both 6 and 12 months post-surgery compared to those who underwent open surgery. The mean CTC count for the laparoscopic group at 6 months was significantly higher than that of the open surgery group (*p* = 0.05), and this trend persisted at 12 months (*p* = 0.062).

## Discussion

This prospective study demonstrates that persistent and rising CTCs after curative hepatectomy strongly predict early recurrence and poor survival in HCC. While CTCs were detectable in nearly half of patients preoperatively, their prevalence increased to 95% at 6 months and 100% at 12 months, suggesting that surgical resection does not eliminate all tumor cell dissemination and that CTCs may reflect minimal residual disease (MRD). This finding aligns with the concept of MRD in cancer, where small numbers of cancer cells persist after treatment and may eventually lead to relapse [16, 17]. This is particularly relevant given that HCC has a high recurrence rate, with up to 70% of patients experiencing recurrence within 5 years after curative treatment [18]. We concur with the principle that prior to resection, the level of circulating tumor cells is largely a reflection of the primary tumor burden. However, the prognostic significance of CTCs undergoes a critical transition following surgical resection. In the postoperative setting, the persistence or rebound of CTCs is no longer an indicator of the resected primary mass but rather serves as a direct, real-time biomarker for MRD. This dynamic behavior—a significant decline after effective tumor removal followed by a subsequent rise in patients destined for relapse—precisely aligns with the kinetics of an ideal marker for monitoring recurrence [19]. The heterogeneity in CTC count trajectories among patients highlights the need for personalized monitoring strategies in HCC management. The increase in CTCs observed after resection can also be attributed to several factors. Surgical manipulation during resection can lead to the release of tumor cells into the bloodstream, a phenomenon often referred to as ‘surgical stress.’ This process may be exacerbated by the inflammatory response triggered by the surgical procedure, which can promote tumor cell survival and dissemination [20]. Studies have shown that surgical trauma can induce a systemic inflammatory response, characterized by elevated levels of pro-inflammatory cytokines such as IL-6 and tumor necrosis factor-alpha (TNF-α), which may enhance the survival of CTCs in circulation [21].

In this study, we found significantly higher CTC counts in HCC patients compared to both NMLD (*p* = 0.036) and other cancer types (*p* = 0.016) before surgery. This difference persisted after surgery, particularly when compared to other cancer types (*p* = 0.018). These findings support the specificity of our CTC detection method for HCC and suggest that CTCs could be a valuable biomarker for differentiating HCC from other liver conditions. The presence of CTCs in NMLD patients, albeit at lower levels, is an interesting observation that warrants further investigation. Sun et al. reported that preoperative CTC counts were significantly higher in HCC patients compared to those with benign liver diseases, and CTC counts decreased after surgery in most cases [17, 22]. This could potentially be explained by the release of epithelial cells into circulation due to inflammatory processes or early neoplastic changes not yet detectable by conventional imaging techniques [23].

Our findings reinforce the growing evidence that longitudinal CTC monitoring is superior to single time-point assessment. Previous studies have primarily focused on preoperative or immediate postoperative CTCs, which, while informative, may underestimate ongoing dissemination. For instance, Lu et al. reported that preoperative CTC positivity was an independent predictor of early recurrence and poor survival in HCC patients undergoing curative resection [24]. Similarly, Qi et al. found that CTC levels were markedly reduced after curative resection in HCC patients [7]. We observed that persistent CTC positivity after surgery correlated with significantly reduced RFS and OS, highlighting the value of dynamic CTC surveillance for risk stratification. These findings support the utility of CTC monitoring as a valuable non-invasive biomarker for risk stratification and treatment planning in HCC patients, with biological aggressiveness reflected by CTC dissemination appearing to supersede tumor volume in predicting post-surgical outcomes. Moreover, our findings are consistent with previous studies that have demonstrated the association between CTC presence and poor prognosis in HCC [6, 25–28]. Moreover, the longitudinal evaluation of CTC dynamics has revealed that rising CTC levels post-surgery is closely linked to early tumor recurrence. A study by Sun et al. reported that patients with elevated CTC counts after surgical resection had a significantly higher risk of recurrence compared to those with lower counts [27]. This finding underscores the potential of CTC monitoring as a non-invasive method for early detection of recurrence, allowing for timely therapeutic interventions. In our study, we observed similar trends, with CTC levels significantly increasing at 6 and 12 months post-surgery, which correlated with recurrence in a substantial proportion of patients. These findings align with the concept of minimal residual disease, where small numbers of cancer cells persist after treatment and may lead to relapse. The integration of CTC analysis into clinical practice could enhance risk stratification and guide adjuvant therapy decisions, particularly in patients at high risk for recurrence.

Notably, CTC counts showed no correlation with tumor size, resected tumor volume, AFP, or IL-6, indicating that CTCs offer prognostic value independent of conventional biomarkers. This is clinically significant because up to 40% of HCC patients are AFP-negative, and radiologic recurrence is often detected only after biological progression. Incorporating CTC monitoring into postoperative surveillance may therefore facilitate earlier intervention and improved outcomes.

Furthermore, tumor size alone was not significantly associated with OS or RFS in our cohort, underscoring that HCC prognosis is multifactorial. These findings highlight the importance of integrating multiple clinicopathological and biological parameters, such as CTCs, into risk assessment and follow-up strategies, rather than relying solely on tumor dimensions, which, although classically linked to poorer outcomes, do not fully capture disease behavior [29]. However, this lack of correlation is not unprecedented in the literature. Büdeyri et al. found no significant correlation between CTC counts and tumor size in 20 HCC patients [23], neither did a study by Oklu et al. [30]. They proposed that CTC release might be more dependent on the biological characteristics of the tumor, such as its invasiveness and vascular involvement, rather than simply its volume. This hypothesis is supported by research from Cuccurullo et al., who demonstrated that CTC counts were more strongly associated with microvascular invasion (MVI) and tumor differentiation than with tumor size in HCC [31].

Previous studies have demonstrated that MVI is associated with a higher risk of recurrence and poorer overall survival, as it indicates a more aggressive tumor biology and potential for metastasis [31, 32]. While MVI remains a critical marker for predicting recurrence, our data suggest that CTCs may provide complementary information that could enhance prognostic accuracy. In our cohort, the presence of CTCs postoperatively was significantly associated with reduced recurrence-free survival and overall survival, indicating that CTCs could serve as a more dynamic biomarker for monitoring disease progression. The ability of CTCs to reflect real-time tumor dynamics may allow for earlier detection of recurrence compared to static markers like MVI, which are assessed at the time of surgical resection.

Furthermore, the failure to achieve R0 margins is a well-established predictor of recurrence, as residual tumor cells can lead to disease relapse [33]. In our study, half of the R1 resected patients were CTC-positive before the resection. Only 1 patient remained CTC-positive immediately after surgery. The higher appearance of CTC positivity in our study is owed to the limited sample size. The administration of preoperative therapy may also affect CTC levels and recurrence outcomes. While preoperative treatments aim to downstage tumors and improve surgical outcomes, their impact on CTC dynamics can vary. Some studies suggest that preoperative therapy may reduce CTC counts by targeting circulating tumor cells, while others indicate that it may not significantly alter CTC levels [34, 35]. These findings support the growing body of evidence that CTC presence may serve as an independent prognostic marker in solid tumors, including HCC and other gastrointestinal cancers [28, 36–38]. The association between CTC positivity and early recurrence aligns with prior studies showing that disseminated tumor cells can persist and seed micrometastases after surgical resection [38]. These results emphasize the utility of CTC monitoring as a non-invasive biomarker to stratify risk and guide adjuvant therapy decisions.

Moreover, we examined the relationship between CTC levels and two typical serum biomarkers in HCC: AFP and IL-6. While AFP is a well-established biomarker for HCC, its sensitivity can vary, particularly in cases where patients present with low AFP levels or are AFP-negative [39] and its relationship with CTCs appears to be independent, indicating that these markers may reflect different aspects of tumor biology. Several studies showed that CTC counts and AFP levels were independently associated with poor prognosis in HCC patients, suggesting that these two biomarkers may reflect different aspects of tumor biology [33, 35]. The correlation between CTC count and AFP levels has been reported for preoperative HCC cases [40]. However, the lack of correlation between AFP and CTC levels in our analysis suggests that while both markers are elevated in HCC, they may not directly influence each other but rather contribute to a complex interplay of tumor dynamics. Furthermore, the longitudinal evaluation of CTCs in our study highlights their potential role in providing real-time insights into tumor dynamics and recurrence risk, which may not be captured by static markers like AFP. The ability of CTCs to reflect changes in tumor burden more dynamically could enhance patient management strategies, allowing for timely interventions before clinical recurrence is confirmed [41]. IL-6 has been associated with tumor progression and poor outcomes in HCC, but its relationship with CTCs is less well-established. Studies also found that while both IL-6 and CTC counts were elevated in HCC patients compared to healthy controls, there was no significant correlation between the two markers [42, 43]. Elevated IL-6 levels can on the other hand promote tumor progression and metastasis by enhancing the survival of CTCs in the bloodstream. This is achieved through several mechanisms, including the activation of anti-apoptotic pathways and the promotion of epithelial-mesenchymal transition (EMT) [44]. While CTCs originate from the primary tumor, they are not a perfect representation of the bulk lesion but rather a subpopulation selected for metastatic competence. This selection process often involves the EMT, a fundamental change in cell biology that allows for invasion and survival in the circulation. During EMT, tumor cells can downregulate tissue-specific differentiation markers, such as AFP, while gaining mesenchymal traits [45]. Therefore, a discordance between the AFP status of the primary tumor and that of the detected CTCs is biologically plausible and may signify a more aggressive, de-differentiated phenotype poised for metastasis. However, this biological interpretation remains speculative, and technical limitations or the small cohort size could also account for the observed discordance. In our study, we observed that patients with high IL-6 levels often exhibited increased CTC counts, particularly at 6 and 12 months post-surgery (Supplementary Fig. 1). This suggests that IL-6 may create a supportive microenvironment for CTC survival, potentially leading to poor outcomes.

In our study, we observed significant differences in CTC dynamics based on the surgical approach used. The increased CTC levels observed in both groups may reflect the surgical manipulation and the inflammatory response triggered by the procedure, which can lead to the release of tumor cells into the bloodstream. This would align with existing literature that indicates surgical handling can influence CTC dissemination, thereby impacting postoperative outcomes [46]. Wind et al. reported a cumulative percentage of samples with CTCs at significantly higher levels during open surgery (lateral-to-medial approach) compared to a laparoscopic (medial-to-lateral) approach [47]. However, in our study, due to the small number of patients in the laparoscopic subgroup, the trend toward higher CTC counts in laparoscopic vs. open surgery may be due to excessive weighting of individual patients in the laparoscopic group. A separate study will be conducted with an equal number of patients in both the laparoscopic and open subgroups to accurately demonstrate the impact of both procedures. Our results point out the importance of considering surgical technique as a variable in CTC dynamics and highlight the need for personalized monitoring strategies in HCC management. Yet, our results should be carefully analysed since both groups are cumulatively not powered enough owing to low sample size to substantiate such a conclusion.

This study has attrition-related limitations such as the sample size and the follow-up period. The high postoperative CTC positivity rate likely reflects a combination of factors: the sensitivity of our CD45⁻/CD146⁺/ASGPR⁺ flow cytometry method, the inclusion of nearly half of patients with intermediate or advanced BCLC stages, and attrition bias at later time points, where mostly high-risk patients remained. Control analyses using healthy donors confirmed assay specificity, suggesting that the observed positivity is biologically meaningful rather than an artifact. Yet, a further molecular characterization of CTCs such as genomic and transcriptomic profiling could have provided sophisticated insights into tumor`s biological behaviour shedding light on the heterogenous longitudinal CTC dynamics. Also, we restricted our primary analysis to the immediate postoperative time point, as this interval offered the most complete dataset and allowed for statistically interpretable comparisons. Although specimens were also obtained preoperatively and during later follow-up visits, the limited number of evaluable samples and missing data at these time points reduced analytical power. We are continuing to enrol patients and collect longitudinal samples, which will support future analyses aimed at elucidating biomarker trajectories across multiple postoperative intervals. Given the limited number of participants in our study, we acknowledge that these factors may limit the robustness of our conclusions. However, our findings still provide valuable insights into the prognostic relevance of CTCs in HCC patients undergoing surgical resection. We are actively collecting additional samples and planning an expanded, adequately powered study designed specifically to support robust multivariable and time dependent analyses. We recommend that future studies with larger multicenter cohorts further investigate the interplay between these variables and their collective impact on recurrence and CTC dynamics.

## Conclusions

This study highlights the prognostic significance of CTCs in HCC following curative hepatectomy. Persistent or rising postoperative CTCs were strongly associated with early recurrence and poorer survival, underscoring their potential as a non-invasive biomarker of minimal residual disease. Yet, in our study the 12-month CTC detection of 100% was influenced by attrition and selection bias and should not be directly generalized to all surgically treated HCC patients. Therefore, our findings should be confirmed in larger cohorts with longer follow-up periods. Longitudinal CTC monitoring could enhance current follow-up strategies by identifying high-risk patients earlier, offering a therapeutic window before overt metastasis occurs. Moreover, CTCs may represent a future target for adjuvant interventions aimed at preventing recurrence. Larger, multicenter studies with extended follow-up are warranted to validate these findings and establish CTC-guided surveillance and treatment algorithms for HCC.

## Supplementary Information

Supplementary Material 1.

## Data Availability

The datasets used and analyzed during the current study are available from the corresponding author on reasonable request.

## Abbreviations

HCC: Hepatocellular carcinoma
RFS: Recurrence-free survival
OS: Overall survival
CD: Cluster of differentiation
ASGPR: Asialoglycoprotein receptor
CT: Computed tomography
MRI: Magnetic resonance imaging
AFP: Alpha-fetoprotein
CTC: Circulating tumor cell
FACS: Fluorescence-activated cell sorting
IF: Immunofluorescence
BCLC: Barcelona Clinic Liver Cancer
NMLD: Nonmalignant liver disease
EDTA: Ethylenediaminetetraacetic acid
PBMC: Peripheral blood mononuclear cell
PBS: Phosphate-buffered saline
BSA: Bovine serum albumin
MCAM: Melanoma cell adhesion molecule
DAPI: 4′,6-diamidino-2-phenylindole
MASH: Metabolic-associated steatohepatitis
