# Supplementary material for: Longitudinal monitoring of circulating tumor cell dynamics for potential prediction of early recurrence and clinical outcomes after curative resection of hepatocellular carcinoma: a pilot study
Source: BMC Cancer. 2026 Jan 29;26:289. doi: 10.1186/s12885-026-15638-7 (PMC12924575; doi:10.1186/s12885-026-15638-7)
Supplement: Supplementary file 1 — Supplementary Material 1. [file 12885_2026_15638_MOESM1_ESM.docx]

**SUPPLEMENTARY DATA**

Supplementary Data are available at xxx: Supplementary Figure 1: Identification of CTCs in HCC patients via immunofluorescence. Supplementary Figure 2: Scatter plots showing the relationships between AFP (x-axis) and IL-6 (y-axis) at three time points after surgery (immediate, 6 months, and 12 months), with CTC counts represented by both point size and color intensity.


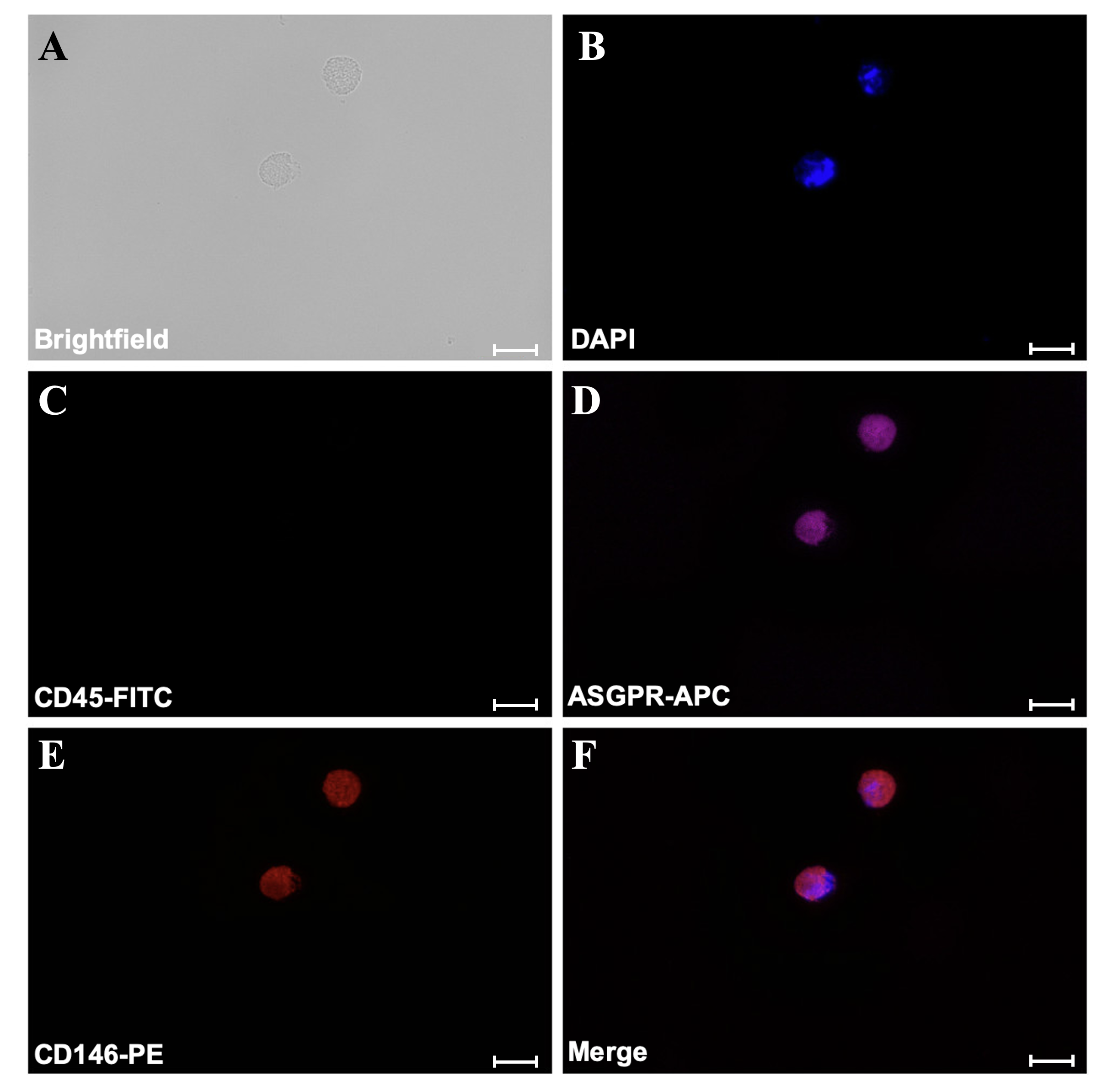


**Supplementary Figure 1.** Identification of CTCs in HCC patients via immunofluorescence by A) Brightfield, B) DAPI C) CD45-FITC, D) ASGPR-APC, E) CD146-PE, F) Merge. Scale bar: 50 μm.


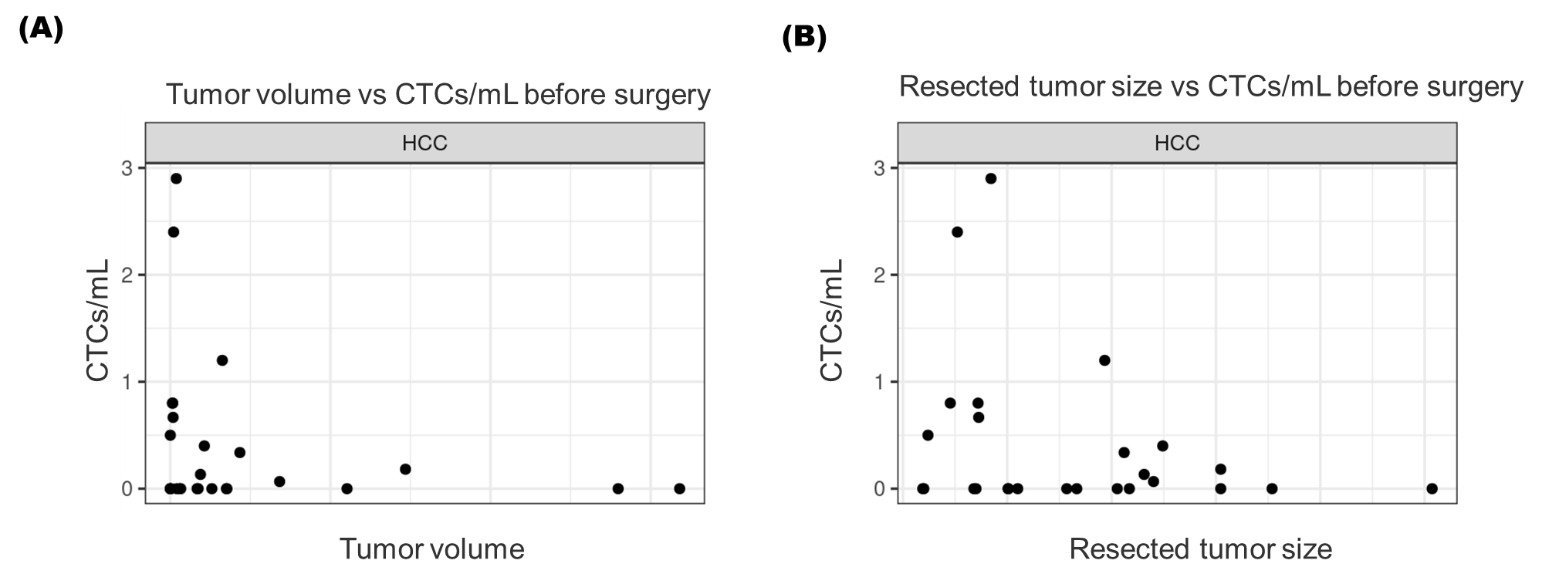


**Supplementary Figure 2.** Correlation between CTC levels before surgery and tumor characteristics in HCC patients. The analysis examines correlations between CTCs/mL before surgery and tumor volume (A) and resected tumor size (B).


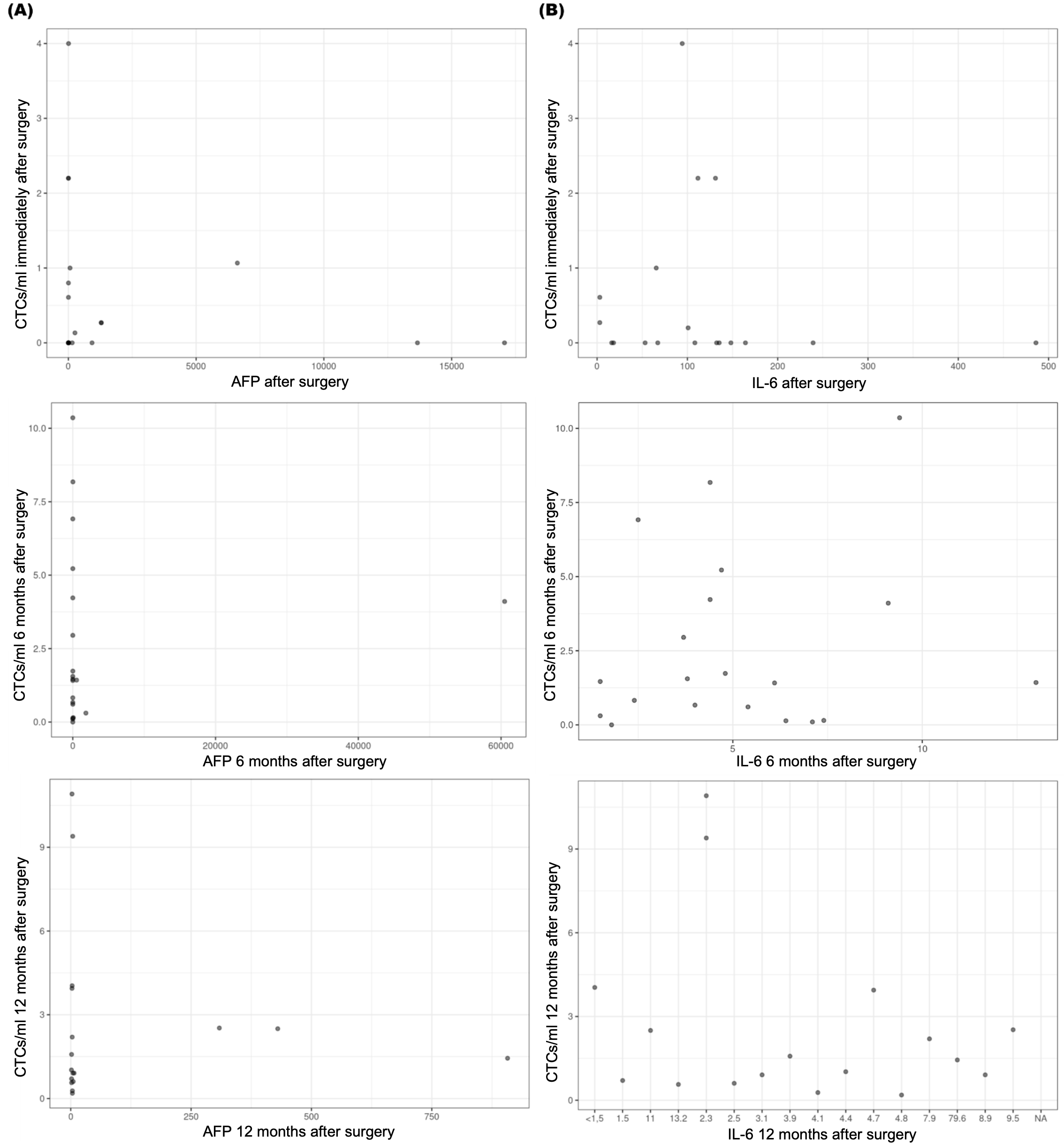


**Supplementary Figure 3.** Scatter plots examining correlations between CTC levels and two biomarkers—AFP (Alpha-fetoprotein) and IL-6 (Interleukin 6)—at three time points following surgery: immediately after surgery, 6 months, and 12 months after surgery. Figure A: Correlation between alpha-fetoprotein (AFP) levels and CTC counts. (Top) Immediately after surgery, (Middle) 6 months after surgery, (Bottom) 12 months after surgery. Figure B: Correlation between interleukin-6 (IL-6) levels and CTC counts. (Top) Immediately after surgery, (Middle) 6 months after surgery-, (Bottom) 12 months after surgery.


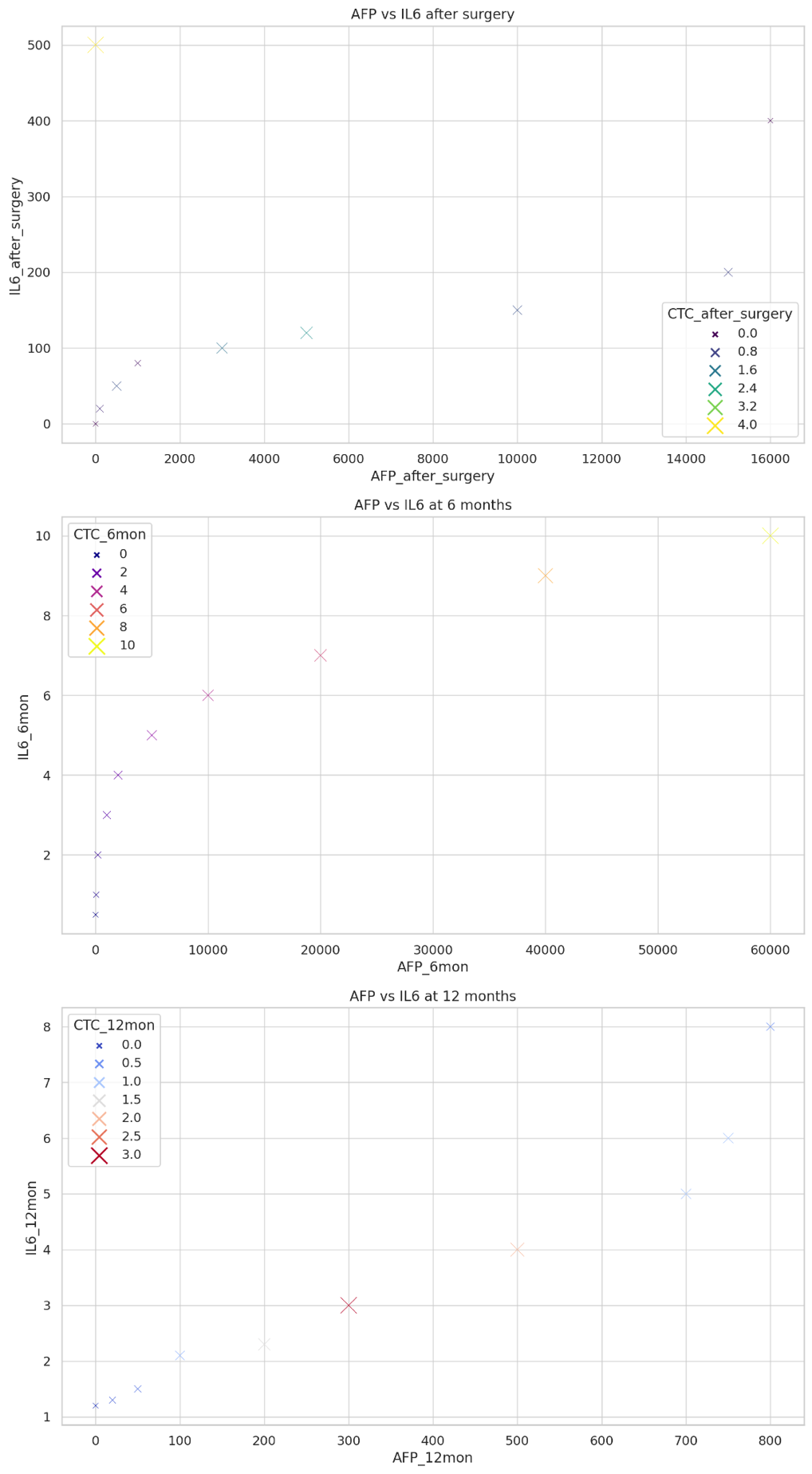


**Supplementary Figure 4.** scatter plots showing the relationships between AFP (x-axis) and IL-6 (y-axis) at three time points after surgery (immediate, 6 months, and 12 months), with CTC counts represented by both point size and color intensity.


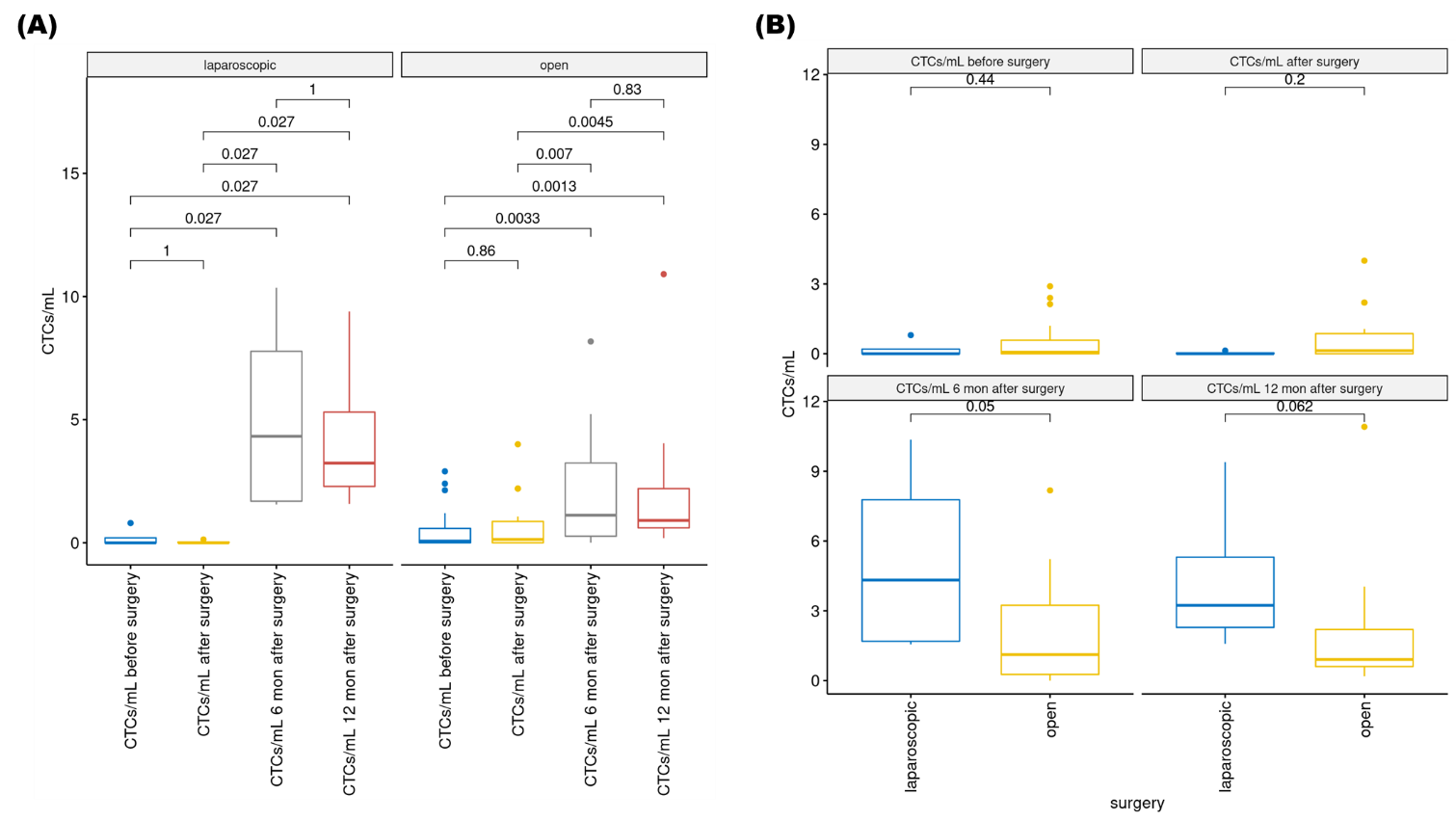


**Supplementary Figure 5.** Comparison of CTCs (CTCs/mL) at four time points—before surgery, immediately after surgery, 6 months post-surgery, and 12 months post-surgery—between patients undergoing laparoscopic (left panel) and open (right panel) surgery. Each boxplot represents CTC distribution at the corresponding time point. Statistical comparisons (p-values) were calculated using pairwise tests within each surgical group. (A) Intra-group comparisons show a statistically significant increase in CTCs over time within both surgical groups, particularly at 6 and 12 months post-surgery.

(B) Inter-group comparisons reveal no significant differences at baseline or immediately after surgery (p = 0.44 and 0.2 respectively), but CTC levels trended higher in the laparoscopic group at 6 months (p = 0.05) and 12 months (p = 0.062).
